# Supplementary material for: The genome sequence of Quercus cerris L., 1753 (Fagales: Fagaceae)
Source: Wellcome Open Res. 2026 Jun 18;11:368. [Version 1] doi: 10.12688/wellcomeopenres.26783.1 (PMC13332380; doi:10.12688/wellcomeopenres.26783.1)
Supplement: Peer review history

# Reviewer report: Reviewer response for version 1

**DOI:** 10.21956/wellcomeopenres.29489.r158701

**Published:** 2026-08-25

**Authors:** Roberto Biello

**Affiliations:** 1 University of Copenhagen Globe Institute (Ringgold ID: 552766), Copenhagen, Capital Region of Denmark, Denmark

This manuscript presents a high-quality, chromosome-level reference genome assembly for *Quercus cerris*, an ecologically and economically important species and a valuable addition to the Darwin Tree of Life (DToL) project. Methods follow standard DToL procedures, and the resulting assembly is highly contiguous, with 99.89% of sequence anchored into 12 chromosomal pseudomolecules and a BUSCO completeness of 98.2%.

 The manuscript presents different genome size estimates across methods, with 1.6 Gb from GenomeScope2.0, 0.96 Gb from flow cytometry, and 0.77 Gb from the primary contig assembly. I understand that estimates from different methods do not always converge, for various reasons, but it would be helpful if the authors could comment on this discrepancy.

Are sufficient details of methods and materials provided to allow replication by others?

Yes

Is the rationale for creating the dataset(s) clearly described?

Yes

Are the datasets clearly presented in a useable and accessible format?

Yes

Are the protocols appropriate and is the work technically sound?

Yes

Reviewer Expertise:

Genomics, Genome assembly, Population genomics

I confirm that I have read this submission and believe that I have an appropriate level of expertise to confirm that it is of an acceptable scientific standard.

# Reviewer report: Reviewer response for version 1

**DOI:** 10.21956/wellcomeopenres.29489.r158695

**Published:** 2026-07-06

**Authors:** Peng-Fei Ma

**Affiliations:** 1 Chinese Academy of Sciences, Kunming, China

This study presents the first chromosome‑level reference genome for Turkey oak ( *Quercus cerris*), with excellent assembly statistics (99.89% anchored to 12 pseudomolecules and a high BUSCO score of 98.2%). The plastid and mitochondrial genomes were also assembled and annotated. In short, the genomics resource will be valuable for studying the Fagaceae evolution and forestry breeding in future.

Are sufficient details of methods and materials provided to allow replication by others?

Yes

Is the rationale for creating the dataset(s) clearly described?

Yes

Are the datasets clearly presented in a useable and accessible format?

Yes

Are the protocols appropriate and is the work technically sound?

Yes

Reviewer Expertise:

Plant genomics and evolution

I confirm that I have read this submission and believe that I have an appropriate level of expertise to confirm that it is of an acceptable scientific standard.

# Reviewer report: Reviewer response for version 1

**DOI:** 10.21956/wellcomeopenres.29489.r158692

**Published:** 2026-07-03

**Authors:** Himanshu Saini

**Affiliations:** 1 Adama Science and Technology University, Adama, Ethiopia

This manuscript presents a high-quality chromosome-level genome assembly for *Quercus cerris*, an ecologically and economically important oak species. The assembly is highly contiguous, with 99.89% of the genome anchored into 12 chromosomal pseudomolecules, demonstrating excellent assembly quality. The inclusion of complete mitochondrial and plastid genome assemblies further enhances the value of this genomic resource.

 The annotation identifying 25,805 protein-coding genes provides a strong foundation for future studies on oak biology, evolution, adaptation, and conservation. As part of the Darwin Tree of Life Project, this work represents an important contribution to the growing collection of high-quality reference genomes for the biodiversity of Britain and Ireland.

 Overall, the manuscript is well presented, the methodology appears robust, and the resulting genome assembly constitutes a valuable resource for the plant genomics community. The data will facilitate comparative genomics, population genetics, evolutionary biology, and breeding research in oaks and related species. I recommend this manuscript for indexing with no major concerns.

Are sufficient details of methods and materials provided to allow replication by others?

Yes

Is the rationale for creating the dataset(s) clearly described?

Yes

Are the datasets clearly presented in a useable and accessible format?

Yes

Are the protocols appropriate and is the work technically sound?

Yes

Reviewer Expertise:

Plant Breeding and Biotechnology

I confirm that I have read this submission and believe that I have an appropriate level of expertise to confirm that it is of an acceptable scientific standard.

# Reviewer report: Reviewer response for version 1

**DOI:** 10.21956/wellcomeopenres.29489.r158699

**Published:** 2026-06-29

**Authors:** Nils Stein

**Affiliations:** 1 Leibniz Institute of Plant Genetics and Crop Plant Research (IPK), Seeland, Germany

The study reports the genome of Q. cerris, a species relevant to the UK DToL project. Methods are following standard procedures established for the DTol project and data metrics look convincing and of high quality, hence the study reports a haploid assembly exceeding the minimal requirements set as standard in DToL.

 Genome size is reported by different methods, and I recommend adding more conclusive comments on the proposed final haploid genome size.

 In the Genome Sequence Report/Sequence Data paragraph a haploid genome size of 1.6 Gb is reported based on the GenomeScope2.0 analysis. Flow cytometry indicated 0.96 Gb and primary contigs comprise 0.77 Gb. I suppose that the GenomeScope 2.0 value is in fact indicating a diploid genome size estimate. It would be useful to report also the size of the alternate contigs and give a cumulative length of both haplotypes, however, due to low heterozygosity the cumulative size of the alternate haplotype is likely to be smaller.

 Gene numbers are relatively low compared to other species. Any comment?

 A summary statement of total number of genomes sequenced of species in the Quercus genus could be interesting to the reader.

Are sufficient details of methods and materials provided to allow replication by others?

Yes

Is the rationale for creating the dataset(s) clearly described?

Yes

Are the datasets clearly presented in a useable and accessible format?

Yes

Are the protocols appropriate and is the work technically sound?

Yes

Reviewer Expertise:

Plant Genetics and Genomics

I confirm that I have read this submission and believe that I have an appropriate level of expertise to confirm that it is of an acceptable scientific standard.
